# Research Dissemination Strategies in Pediatric Emergency Care Using a Professional Twitter (X) Account: A Mixed Methods Developmental Study of a Logic Model Framework

**DOI:** 10.2196/59481

**Published:** 2025-06-24

**Authors:** Gwendolyn C Hooley, Julia N Magana, Jason M Woods, Shyam Sivasankar, Lauren VonHoltz, Anita R Schmidt, Todd P Chang, Michelle Lin

**Affiliations:** 1 Children's Hospital Los Angeles Los Angeles, CA United States; 2 University of California, Los Angeles Los Angeles, CA United States; 3 University of California Irvine Irvine, CA United States; 4 University of Colorado School of Medicine Denver, CO United States; 5 Children’s Hospital Colorado Denver, CO United States; 6 Baylor College of Medicine Houston, TX United States; 7 Stanford University School of Medicine Stanford, CA United States; 8 Children's Hospital of Philadelphia Philadelphia, PA United States; 9 University of California, San Francisco San Francisco, CA United States

**Keywords:** social media, research dissemination, knowledge translation, twitter, professional social media account, medical social media, professional twitter, medical education, clinical practice, academic medicine, mixed methods study, qualitative approach, quantitative data, logic model, medical institution, data-driven

## Abstract

**Background:**

Research dissemination is a vital step in bridging the gap between the publication of cutting-edge research and its adoption into clinical practice. Social media platforms like Twitter (rebranded as X) offer promising channels for dissemination, yet research organizations lack clear guidance on establishing a professional social media presence. We present a structured framework based on our research network’s multiyear experience developing a Twitter account for research dissemination.

**Objective:**

This study aimed to provide a roadmap for organizations aiming to create a professional Twitter account for research dissemination.

**Methods:**

This was a mixed methods study analyzing the Pediatric Emergency Care Applied Research Network (PECARN) Twitter team’s 4-year experience (2020-2023) with building a social media account. Using the nominal group technique qualitative approach, we recorded insights from the 6 team members’ experiences in a round-robin fashion until response saturation. In addition, we analyzed internal Slack (Slack Technologies) communications to identify key developmental events. Together, these were then prioritized by consensus to elucidate key developmental events that enhanced both social media and scientific engagement. This process was informed by quantitative data from Twitter performance metrics and Altmetric Attention Scores for journal publications collected over a 39-month period. Together, these elements informed the design of a logic model framework.

**Results:**

The nominal group technique generated 63 thematic statements which included issues such as organizational structure, content strategy, technologies, analytics, organizational priorities, and challenges. These statements coalesced into the 7 domains (priorities, assumptions, inputs, outputs, outcomes, and external factors) that comprise the logic model. Inputs included organizational support (eg, executive-level champion and funding), specialized personnel (eg, content writer and analytics manager), and operational technologies (eg, communications and data analytics tools). Outputs encompassed targeted activities, such as engaging with other Twitter accounts, publishing high-quality tweets highlighting scholarly work, and developing a dynamic operations manual for the Twitter team. Outcomes were measured through tweet metrics, account analytics, and article-level impact scores.

**Conclusions:**

Our logic model roadmap, based on our practical multiyear experience and data-driven strategies, can serve as a guide for research organizations or medical institutions aiming to incorporate Twitter or other social media platforms for research dissemination.

## Introduction

Despite technological advances dramatically increasing the rapidity with which new information becomes available, studies demonstrate that there is a significant lag between the publication of new research and its adoption into clinical practice [1,2]. Social media holds the potential to reduce this lag by promptly informing clinicians and policy makers about the latest practice-changing literature [3,4]. In particular, Twitter (now rebranded as X, though “Twitter” is used in this article given its widely recognized name) has over 600 million monthly active users [5] and has gained traction across clinical subspecialties, journals, and academia for research dissemination [6-8]. Having a Twitter presence is becoming increasingly important as a means to reach target audiences and affect bibliometric markers of research impact. As academic Twitter usage has increased, studies have identified characteristics that make tweets more effective from a research dissemination perspective, such as the incorporation of graphics and campaigns coinciding with target events [9-11]. While numerous studies have outlined content strategies to boost engagements and impressions in research dissemination, there is limited guidance for research organizations and medical institutions on the key steps to building and maintaining a professional Twitter account for research dissemination [12-14], such as the personnel needed to manage a social media account and technical resources required in addition to the platform itself.

The Pediatric Emergency Care Applied Research Network (PECARN) is a US-based, federally funded, multi-institutional consortium specializing in pediatric emergency research, with over 200 peer-reviewed publications on improving pediatric emergency care. PECARN is funded by the Health Services and Resources Administration, a governmental agency dedicated to improving access to health care, particularly in vulnerable communities. In 2019, PECARN renewed federal funding with an added focus on information dissemination. In partnership with Academic Life in Emergency Medicine (ALiEM), an education organization with expertise in social media–based scholarship and dissemination, a joint decision was reached to launch a professional Twitter account (@PECARNteam) for showcasing PECARN’s publications. This led to the formation of the PECARN Twitter team, which began posting tweets in 2020. The team was ultimately composed of 8 members including 2 content writers, 2 peer reviewers, 2 account monitors, an analytics manager, and a graphic designer. Drawing from the team’s experiences along with the best practices from the business, marketing, and publishing sectors, we launched and grew the professional account through iterative design.

In this report, we share the PECARN Twitter team’s experiences, reflections, and analytics over a 4-year period (2020-2023) to establish a professional social media presence for a national research organization. We identify key components for the creation and maintenance of an organizational social media account, supported by quantitative data from the study period. Our objective is to offer a structured roadmap in logic model format [15] for research organizations, national societies, and medical institutions aiming to accelerate research dissemination using a professional Twitter account.

## Methods

### Overview

We used a convergent mixed methods design to encapsulate the Twitter team’s experience during 2020-2023 [16]. For the qualitative strand, we used the nominal group technique (NGT) to elicit as many opinions and recollections of key events, developments, and challenges, from each Twitter team member; our goal was to build consensus among the Twitter team members about which essential steps were undertaken to build the PECARN Twitter account [17]. In addition, we analyzed our team’s 4-year Slack (Slack Technologies) communication history to extract significant events and decisions, ensuring no major events were overlooked. For the quantitative strand, we performed an analysis of Twitter metrics and the Altmetric Attention Score. We combined these data sources to create a logic model, which outlines a roadmap for institutions and research organizations aiming to build a professional Twitter account for research dissemination.

### Ethical Considerations

This study received exempted approval from the Institutional Review Board at Children’s Hospital Los Angeles. An information sheet described the research purpose and NGT. Participants understood that their identities, which were publicly available on PECARN and ALiEM websites, could not be fully anonymized. They could email questions to the principal investigator before participating or opting out. Participation implied consent to using responses for the study. No participants were compensated for participating in the study. The NGT occurred on a secure internet platform, with transcripts stored on a password-protected laptop.

### Qualitative Methods

#### NGT

The NGT is a consensus-building technique led by a facilitator, whereby a group of 5-9 participants answer a discussion prompt and then participate in a structured brainstorming session [17,18]. The purpose of our NGT was to compile a comprehensive list of the essential steps taken to build the PECARN Twitter account. In total, 6 core members of the PECARN Twitter team gathered on an online videoconference platform over the course of 2 hours with 2 external facilitators (ARS and TPC) with NGT expertise. The session omitted 2 recently added team members due to their limited involvement over the study period.

Our prompt was as follows: “Please describe, in as much detail as possible, the specific activities, events, and milestones that happened while creating the current PECARN Twitter account, including barriers encountered along the way.” The language of the prompt was crafted through iterative feedback from 3 external qualitative researchers to ensure clarity, appropriateness, and scope. After being presented with the prompt, participants were first given 5 minutes to independently record their ideas. Subsequently, participants participated in a round-robin fashion, each sharing ideas in turn until all new ideas were exhausted. The final phase of the NGT entailed a group discussion allowing individuals to clarify any concepts or ideas. In contrast to typical NGT brainstorms, participants did not rank the ideas. Instead, we grouped related concepts together and condensed similar ideas into representative statements. The overarching themes laid the groundwork for what would eventually become the logic model.

#### Slack Analysis

Slack is a real time private messaging and collaboration platform employed by the Twitter team to assist with running the PECARN Twitter account. It served as the primary means of communication during our study. All conversations are saved to the platform in chronological order with dates and timestamps, providing an objective record of major developments. To avoid overlooking key events, 2 of the researchers who were part of the Twitter team reviewed the conversations and subthreads over the course of our study period to generate a timeline of events. The timeline was reviewed and revised several times with input from the Twitter team members. These events were then compared with the events recalled in the NGT. Events not mentioned in the NGT but felt to be crucial to the development of a social media account for dissemination, such as the incorporation of content creators and analytics managers, were then included in the development of our logic model. Using author consensus, events were deemed crucial if they led to a key development in the account, such as creating a new role fundamental to the execution of the account, initiation of a new activity that became routinely included in our content, or led to new guidelines surrounding the management of the account.

### Quantitative Methods

#### Tweet-Level Analysis

We manually collected descriptive data on individual tweet characteristics during a 39-month period (May 2020-August 2023). Tweets are posts on the Twitter platform limited to 280 characters and can include graphics, videos, and links. We performed a multiple linear regression analysis (Microsoft Excel v16.81) using data from December 2020-August 2023 to determine whether 6 tweet characteristics influenced impressions (view count of a tweet) and engagement (user interactions with a tweet including retweets, likes, replies, poll votes, media views, mentions, and clicks on URLs or hashtags).

We excluded the May-November 2020 period because of the “honeymoon period” of starting a Twitter account, where the promotion and novelty surrounding a Twitter account generate a spike and wide variability in engagement [19]. In addition, this was the beginning of the COVID-19 pandemic, which may have altered the online presence of health care professionals. Based on existing marketing literature [12,13,20], 6 independent variables—account tagging, emojis, graphics (image or video), hashtags, polls, and URL links—were selected. Each variable was binary and coded with a 0 (absent) or 1 (present) for each tweet. All 6 independent variables were entered simultaneously in the multiple linear regression model as binary dummy variables. We then calculated standardized beta coefficients (β) post hoc using the formula: β=b×(SD_predictor÷_SD_outcome_) where b is the unstandardized coefficient, SD_predictor_ is the standard deviation of the tweet characteristic (binary variable), and SD_outcome_ is the standard deviation of impressions or engagements. The SD_predictor_ was derived from tweet characteristic frequencies using 
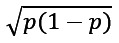
 for binomial distributions. Using a standardized beta coefficient–enabled direct comparison of effect sizes across variables measured on different scales.

Before this analysis, we conducted a multicollinearity check using a correlation matrix (Multimedia Appendix 1). We also used Seaborn to assess linearity and homoscedasticity [21]. These analyses confirmed that our independent variables met the necessary assumptions required for linear regression.

#### Twitter Account Analysis

We monitored monthly account-level data for follower counts from May 2020 to August 2023, using the Twitter analytics services and the Fedica software. We also assessed follower demographics by manually categorizing the Twitter followers by profession (any physician, emergency physician, emergency medical service [EMS] provider, other health care worker, non–health care worker, or medical organization) as well as domestic or international. The follower list was downloaded in April 2021 and October 2022 by 5 PECARN research associates, with codification ambiguities adjudicated by consensus among the team.

#### Altmetric Attention Score Analysis

The Altmetric Attention Score, a newer bibliometric measure in academia to complement traditional citation counts, gauges a journal article’s digital reach and attention [22]. Although tweet mentions of the journal article correlate with increased citation counts [13,22-31], citation counts experience a publication lag. The Altmetric score instead is a weighted, composite, real-time measure of the amount of attention a publication has received through various outlets, including Twitter, blogs, news media, and policy documents [22,32]. The Altmetric website asserts that tweets mentioning a journal article’s Digital Object Identifier (DOI) or PubMed URL will positively raise the Altmetric score, although the exact calculation is not publicly available. To assess this association between our Twitter activity and a journal article’s attention, we conducted an exploratory analysis of the Altmetric score over a 9-month period (August 2020-April 2021). Specifically, for each PECARN publication featured on our Twitter account, we manually recorded the Altmetric score directly from the Altmetric website at 2 specific time points, that is, Sunday immediately before posting the Monday tweet about the article and again the next Sunday (1 week later). This before-and-after approach allowed us to capture any changes in the Altmetric score potentially attributable to our tweets. These scores were publicly available and free on the Altmetric website only for the current day.

### Logic Model

A logic model is a visual way to present the relationships and resources required to execute a program [33]. We derived a logic model using abductive reasoning based on the qualitative NGT and quantitative analytic data to illustrate the relationships between inputs (essential resources), outputs (activities and audience), and outcomes (direct result of activities) in managing a professional Twitter account for research dissemination [15,34]. We cross-referenced our final logic model with the W.K. Kellogg Foundation Logic Model Development Guide’s checklists to ensure comprehensive coverage and internal consistency of all essential elements in the framework [33]. To strengthen the model, we examined our underlying assumptions and unforeseen external factors. Because of the complex, intersecting relationships of the NGT data, any concepts that could be categorized into more than 1 logic model domain were adjudicated by the authorship team.

To enhance generalizability, the final proposed logic model maintains the core elements of the original framework but uses broader terminology. For logic model elements that were not directly addressed in the NGT, such as personnel or audience, we referenced our internal Slack communications and the PECARN website.

## Results

### Overview

Overall, the NGT generated 63 unique thematic statements, which addressed issues such as organizational structure and management, content strategy and outreach, technologies, analytics, organizational priorities, and challenges. These concepts were grouped based on the logic model domains (Textbox 1). Of note, some statements were slightly reworded in Textbox 1 to protect proprietary and private content.

**Figure 1:**
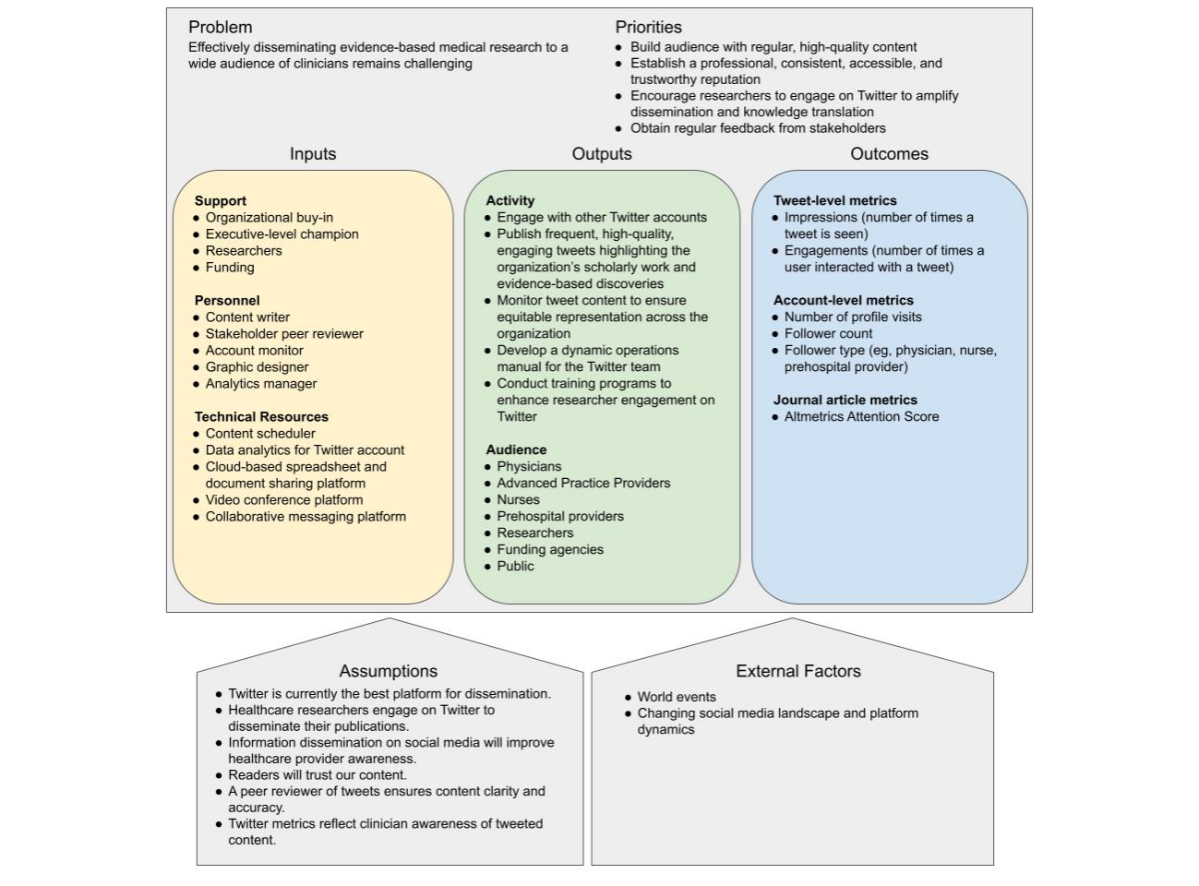
Proposed logic model for implementing a professional organizational Twitter account for research dissemination.

We then transformed our project-specific findings into a more generalizable logic model to provide professional organizations with a roadmap for research dissemination using Twitter (Figure 1). For instance, external factors beyond our organizational control that impacted the Twitter campaign included the COVID-19 pandemic and Elon Musk’s acquisition and rebranding of the platform in 2022. These 2 specific scenarios were generalized into “world events” and “changing social media landscape and platform dynamics.” The following paragraphs provide a deeper exploration of the assumptions, inputs, outputs, and outcomes with each section outlining our general recommendations and our team’s experiences.

**Nominal group technique concepts categorized into the logic model domains with each unique concept designated by a unique letter code.**

**Situation**

- Pediatric Emergency Care Applied Research Network (PECARN) contracts Academic Life in Emergency Medicine (ALiEM) for social media management (M)
- Integration of interdisciplinary and intergenerational members (BD)
- PECARN Champion (BE)
- Team lead with expertise and connections (AI)
- Creation of a dissemination working group (F)
- Securing funding for dissemination (AO)
- Recruitment of Twitter-savvy users to the team (A)
- P3 Project (1)

**Priorities**

- Preemptive education on responding to negative feedback (O1)
- Navigating nodes with equity or balance (AR)
- Getting the word out within PECARN equitably (BF)
- Presentations to the PECARN Steering Committee including updates to increase awareness (BG)
- Encouraging researcher engagement (AT, V1)
- Becoming aware of new publications (AS)
- Anticipating events or bursts of demand (AE)
- Establishing credibility on Twitter (AP)

**Assumptions**

- Peer review of tweets for credibility or trustworthiness (H)
- No gold standard for analytics makes measuring impact or effectiveness difficult (N)

**Inputs**

- Incorporation of best Twitter practices from the marketing world for visibility and engagement (AQ)
- Working with and managing expectations from stakeholders (W)
- Balancing researcher involvement (B, BH)
- Organized structure and systematic review process for creating and editing tweets before publication (E)
- Survey to authors for impending publications (AY)
- Having multiple people making content to cover for each other (AL)
- Reach out to authors for live tweeting (BC)
- Regular meetings, feedback incorporation, and strategic development (R)
- Acquisition of external graphics designer (G)
- Selection of software and metrics (J)
- Learning to work with existing technologies for scheduling and publishing (AF)
- Sudden unexpected team member change (U)

**Outputs**

- Extracting key teaching points from top PECARN articles into tweet (X)
- Tweet frequency established at the same time each week (3 times per week) (AH)
- Tweet visual structure: Intentional or strategic polls, emojis, bullet points, and multimedia such as images and videos (AA, AB, AC, C, D)
- Linking to open-access and other websites, blogs, and source material (AZ)
- Threading continuous tweets (BB)
- Use of hashtags including tagging other existing conferences for more visibility (AN)
- Researcher profile highlights including narrative story to provide sufficient content (BA)
- Priority list of timely content for tweets such as newly published PECARN studies with icons to assist readers (AG)
- Iterative operation manual with protocols and policies to align with stakeholders and unexpected obstacles (S, T, Y)
- Developing approach to unexpected events and outside-of-scope requests (O, AD, AM, AU)

**Outcomes**

- Development of analytic metrics with dashboard monitoring (Q)
- Increasing and broadening followership for Twitter account (AK, AX, K, P)
- Tracking pre- and post-Altmetric scores following tweets (AJ)
- Understanding how PECARN account interacted with other accounts (AV)
- Researching and categorizing follower characteristics to assess who we were reaching (AW)

**External factors**

- COVID-19 pandemic at launch led to concerns over delay (L)
- Flexibility and responding to events occurring on Twitter (Y1, AE1)

### Assumptions

#### Recommendation

Organizations creating a social media account for research dissemination must assume their chosen platform is widely used by the target audience and that sharing content will effectively disseminate research. They must also presume that selected metrics serve as the best surrogate measures reflecting dissemination and clinician awareness, despite the inherent challenges in measuring social media impact.

#### PECARN Experience

In building our logic model, we made several assumptions. We prioritized Twitter over other popular social media platforms because Twitter has been a dominant platform in academic medicine, used by health care professionals, researchers, institutions, organizations, and academic journals [6-8,12]. The platform has many advantages for dissemination including accessibility, reach, conversational tone, and communal nature which allows for a dynamic exchange of ideas within the medical community. Twitter’s format, with its 280-character limit, makes it easily digestible and well-suited for busy health care professionals.

Another assumption we made was that a pre-tweet peer review process ensured the dissemination of trustworthy, unambiguous, and accurate content. In the era of misinformation, this vetting process ensured our tweets could better withstand academic scrutiny and aligned with the values of PECARN. We also assumed our use of a formal Twitter handle which included our organization’s name (@PECARNteam), prominent academic publications, and professional tone would engender trust.

Finally, we assumed the Twitter metrics of impressions and engagement were the best surrogate measures to reflect clinician awareness of the PECARN articles and their application toward patient care. We also explored the impact our tweets may have had on each featured article’s Altmetric Attention Score as another indirect measure of impact that organizations may use.

### Input: Support

#### Recommendation

Securing endorsement from leaders and stakeholders is a fundamental starting point. Stakeholders may include researchers, an internal communications department, funding organizations, and institutions that have a vested interest in the research being disseminated. Ideally, this includes an executive-level champion who can advocate for the social media team.

#### PECARN Experience

In 2017, a senior PECARN researcher met with the founder of an external organization, (ALiEM), who had over 10 years of Twitter experience in the health professions education domain. Their interests aligned when PECARN’s federal funding increasingly valued research dissemination since ALiEM’s mission was to accelerate knowledge translation using social media. Continued discussions ultimately led to a funded partnership whereby ALiEM would serve as PECARN’s Twitter account manager, with peer review oversight by designated PECARN members.

### Input: Personnel

#### Recommendation

In total, 5 integral roles form the foundation of a Twitter research dissemination team. A content writer crafts tweets and schedules content for publication. This person also serves as the team lead, collaborating with the peer reviewer (verifies tweet accuracy and ensures alignment with organizational mission), graphic designer (optimizes visual elements), account monitor (engages with other Twitter accounts), and analytics manager (monitors and reports account metrics). All roles should ideally have previous Twitter experience.

#### PECARN Experience

In building our 8-member Twitter team, deliberate consideration was given to the group composition, reflecting a strategic blend of expertise and flexibility. Of the total, 2 were content writers, who were physicians with an established Twitter presence focusing on emergency medicine and pediatric education. The decision to enlist 2 content writers was intentional, as they had full-time physician jobs competing for their time. Having duplicate writers ensured consistency despite scheduling constraints and individual availability. The 2-writer model served as a safeguard against disruptions, providing a safety net for when a content writer departed the project, requiring the rapid onboarding of a new writer. This redundancy ensured project momentum, content quality, and institutional memory.

For similar reasons, duplicate roles were also created for the peer reviewer and account monitor roles. Peer reviewers, selected from the PECARN organization’s Dissemination Working Group, ensured tweet quality and accuracy. The reviewers also ensured that the studies highlighted by the account equitably represented all of the researchers within the organization and that tweets aligned with PECARN’s mission and values. Account monitors were recruited based on Twitter and research dissemination expertise. They surveilled the Twitter account daily for relevant mentions from other Twitter accounts and replying to, liking, or retweeting their content. A dedicated graphic designer was added 2 years after the launch of the Twitter campaign. This person played a pivotal role in enhancing the visual appeal of the tweets, especially given evidence showing the importance of graphics in promoting engagement [35]. Finally, the analytics manager reported data-driven insights on a monthly basis that informed changes and decisions.

### Input: Technical Features

#### Recommendation

Digital tools are necessary to streamline team communication, documentation, analytics tracking, tweet scheduling, and Twitter account monitoring. These tools should be cloud-based to ensure version control and facilitate asynchronous collaboration among team members, who may be geographically distributed with different work schedules.

#### PECARN Experience

Our Twitter team used digital tools that optimized workflow, efficiency, collaboration, and data-driven decision-making. Slack served as our communication platform, proving more effective than traditional email channels by facilitating real-time collaboration, quick decision-making, and seamless information exchange among team members. Google Sheets (Alphabet Inc) served as a repository for both past and upcoming tweets. This spreadsheet included details such as the responsible team member for each tweet and its current stage (awaiting peer review, graphic design, or publication). The accessibility of this document to the entire team ensured transparency and collaborative tracking of tweet progress. Google Docs (Alphabet Inc) allowed a centralized record of monthly meeting minutes and action plans. In addition, this platform was used to craft an operations manual and style guide. Fedica, a social media management platform, provided data analytics and allowed tweet scheduling. Zoom (Zoom Communications Inc) was the platform for hosting monthly videoconference meetings, allowing for real-time discussions while fostering a sense of community and accountability among team members across locations. Finally, the Twitter mobile app (X Holdings Corp) offered account managers the flexibility to promptly respond to engagements from other Twitter accounts.

### Output: Activity

#### Recommendation

Establishing goals for social media accounts allows for a focused approach in content creation. Growing a following on a professional Twitter account requires a trustworthy, consistent, professional, and engaging presence. Tweets should adhere to the organization’s established content focus and writing style, as well as follow Twitter marketing best practices. Content should be monitored to ensure equitable representation of all individuals and subgroups within the organization. A regularly updated, cloud-based operations manual facilitates easy guideline modifications and streamlines the onboarding process for new team members. To grow an online community and facilitate discussions with intended audiences, conduct training sessions to encourage researchers within the organization to engage directly on Twitter. Iterative evaluation of outcomes data and stakeholder feedback should regularly guide the reassessment of these activities.

#### PECARN Experience

Our overarching goals were to use Twitter to increase awareness of new PECARN publications, provide ongoing education about established PECARN research, introduce evidence-based decision aids for providers to incorporate into their clinical practice, and highlight the PECARN organization as well as its researchers. In the early stages of the PECARN Twitter account, we concentrated on growing our follower base to expand our visibility and reach. We tagged relevant entities, including journals, universities, funding agencies, educational blogs, and prominent physicians in the field.

In conjunction with growing an expanded audience, the Twitter team focused on creating a consistent tweet publication schedule focusing on highlighting both previous and recent PECARN studies. Our group decided to only tweet about full articles, rather than abstracts or preprints, after they were published to ensure the highest quality evidence. To identify new studies, we requested PECARN authors notify us of impending publications. We also configured a PubMed email as well as a Google Alert to notify us of PECARN-related articles. Acknowledging that our Twitter audience might not be aware of previous PECARN research, highlighting older studies was seen as valuable for research dissemination. This approach counters the common trend among journals and news media to concentrate on newer publications. It also allowed us to have a larger pool of content to choose from, allowing us to tweet consistently, even during periods when there were no new publications.

**Figure 2:**
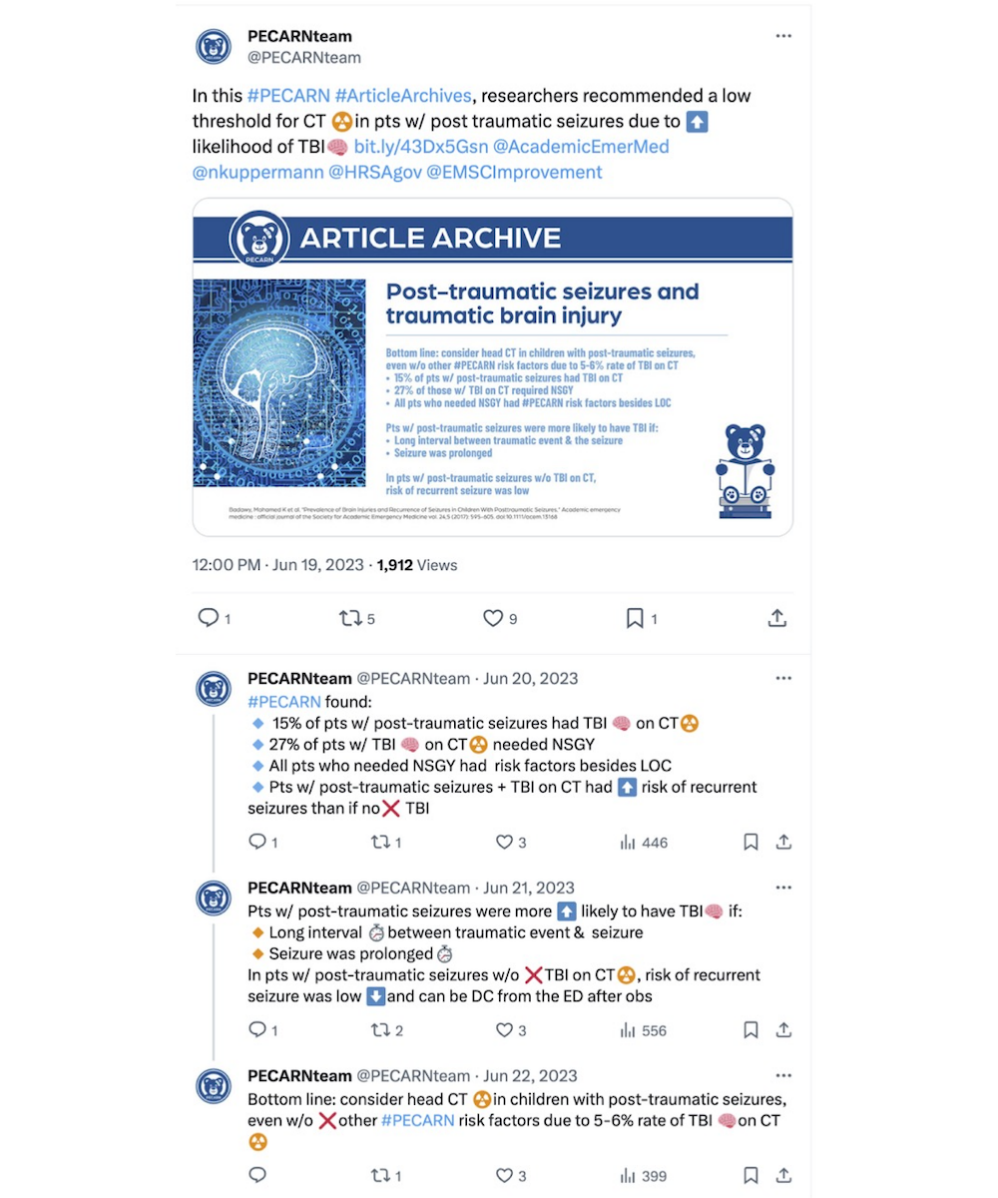
Example of a typical week’s tweet thread discussing a PECARN publication. The first tweet in the series included an image, hashtags, emojis, and a URL link. Subsequent threaded tweets also included bullet points.

Informed by the medical literature, business insights, influencer marketing practices, and our data analytics, our tweet structure aimed to incorporate bullet points, emojis, graphics (image or video), URL links, and hashtags to maximize Twitter engagement and reach [12,13,35-38]. Using Twitter’s threading feature (allowing for a series of connected tweets), each week focused on a single publication. Monday’s tweet introduced the journal article, followed by threaded tweets over the next 5 days, highlighting key results and insights (Figure 2). Tweets were published at the same time of day for consistency.

As the account grew, new strategies were implemented to bolster engagement. In the eighth month after launching the Twitter account (December 2020), polls were incorporated, when possible, to introduce the featured research article of the week. Polls allowed readers to vote on a clinical scenario and see real time polling results. Subsequent threaded tweets reviewed salient teaching points and the significance of the featured journal article. In 2022, a PECARN graphic designer joined the team, incorporating professional graphics into our tweets, allowing for a more detailed description of the article, along with a layout, icons, color scheme, and typeface that better aligned with the PECARN brand. Notably, because the text was embedded in the graphic, we were not constrained to the 280-character tweet limit. During periods of fewer new PECARN publications, selected older studies that we featured on Twitter before 2022 were rehighlighted, incorporating new layout strategies such as professionally designed graphics.

Another goal of the account was the promotion of the PECARN organization and its researchers. To this end, we also monitored content for equitable representation across the organization. Each subgroup and its members had their own unique achievements and perspectives, meriting representation on the organization’s official Twitter account. These tweets were not limited to publications and included national conference presentations, awards, and study achievements. We also occasionally created a series of tweets highlighting specific researchers and their interests.

As we developed our unique style and content strategy, we created an operations manual to help ensure continuity and accountability over time as team members changed and new members joined. This document outlined our tone, structure, content creation workflow, key resources, and general guidelines. It also included a conflict resolution plan, created after a negative reply to one of our tweets regarding concerns about distilling large studies into 280 characters. Furthermore, guidelines on boundary setting outlined which accounts to follow and retweet, as account activity is a direct reflection of the organizational brand. This continuously updated, cloud-based document maintained a shared understanding across time, team members, and stakeholders.

Because of the open community nature of the Twitter platform, researchers can also engage directly with the audience in addressing any questions or amplifying the PECARN tweet messages. Although many PECARN researchers were not active on Twitter at the time of the study, researchers who were active played key roles in retweeting or replying to conversations about their publication. Encouraging researchers to join Twitter through onboarding or training programs may further enhance the reach and visibility of their publications on the platform.

### Output: Audience

#### Recommendation

Every tweet should be tailored with the target audience in mind. This may include health care providers, trainees, researchers, funding agencies, and the lay public.

#### PECARN Experience

The PECARN organization generally aims to reach physicians, nurses, researchers, prehospital providers, and funding agencies with interest or expertise in emergency medicine, pediatrics, and pediatric emergency medicine. Their priority focuses on the US emergency physicians, which includes adult and pediatric emergency physicians as well as their trainees, and EMS providers. Although some tweet content may have been relevant to the lay public, the primary goal was to educate other health care professionals. Thus, tweets were crafted assuming some foundational knowledge of medicine and critical appraisal of clinical research. We distilled each article into relevant key points that might impact practice.

### Outcomes: Tweet-Level Metrics

#### Recommendation

Impressions and engagements are commonly used to measure a tweet’s performance. Additional metrics may be helpful to monitor progress in reaching an organization’s objectives. Native Twitter analytics and third-party data services can provide valuable real-time feedback on tweet performance and audience engagement.

#### PECARN Experience

During May 2020-August 2023, the @PECARNteam Twitter account published 569 tweets, highlighting 99 PECARN publications. We piloted different tweet formats and made data-driven decisions to incorporate at least 1 of the following in each tweet: account tags, emojis, hashtags, graphics, polls, and URL links. A descriptive summary of tweet characteristics and their resultant median number of likes, retweets, replies, impressions, and engagements are reported in Table 1. Notably, polls demonstrated the highest median number of impressions and engagements. Each month, we monitored the best-performing tweets for impressions and engagements, because they provided insights on topics that resonated most with the audience. The median number of clicks for URL links of our featured journal article was 7 (SD 14.9).

We also evaluated whether the above 6 tweet characteristics had an impact on impression and engagement metrics using multiple linear regression analyses. Our model revealed an adjusted *R*^2^=0.22 (impressions) and 0.26 (engagements). Table 2 reports the standardized regression coefficient scores (β), revealing 3 tweet characteristics associated with a significant increase on both impressions and engagements—polls, graphics, and URL links. Notably the inclusion of emojis was associated with a decrease in impressions and engagements.

**Table 1:** Tweet-level analytics associated with the presence of a tweet characteristic (May 2020-August 2023).

Tweet characteristic (number of tweets)	Likes, median (SD)	Retweets, median (SD)	Replies, median (SD)	Impressions, median (SD)	Engagements, median (SD)
Account tagging (n=295)	6 (7.3)	3 (4.9)	1 (0.7)	1008 (2043.3)	29 (58.6)
Emoji (n=347)	3 (6.1)	1(3.8)	1 (0.6)	718 (1697.6)	17 (53.8)
Graphic (n=158)	9 (8.0)	4 (5.3)	1 (0.7)	1608 (2300.1)	47 (74.0)
Hashtag (n=275)	7 (7.8)	3 (5.4)	1 (1.2)	1011 (2155.8)	31 (94.0)
Poll (n=50)	3 (2.4)	2 (1.7)	0 (1.0)	1793 (1733.0)	55 (82.6)
URL link (n=188)	7 (8.1)	3 (5.0)	1 (0.6)	1338 (2272.4)	38.5 (73.2)

**Table 2:** Standardized regression coefficient scores (β) from multiple linear regression analysis for engagement and impressions as influenced by various tweet characteristics for the 461 tweets during December 2020-August 2023.

Tweet characteristic	Impressions (n=461)		Engagements (n=461)	
	Standardized regression coefficient scores (β)	*P* value	Standardized regression coefficient scores (β)	*P* value
Poll	0.243^a^	<.001	0.278^a^	<.001
Graphic	0.225^a^	<.001	0.195^a^	<.001
URL Link	0.198^a^	<.001	0.173^a^	<.001
Hashtag	0.102^a^	.05	0.053	.18
Account Tagging	0.034	.51	–0.018	.64
Emoji	–0.127^a^	.01^a^	–0.073	.06

^a^Coefficient scores with *P*<.05.

### Outcomes: Twitter Account-Level Metrics

#### Recommendation

Tracking longitudinal quantitative data for an organization’s Twitter account can help to identify strategies for growth. Common account-level metrics include follower insights (eg, follower count and demographics), monthly and overall impression and engagement counts, and profile visits (number of times the Twitter profile page is viewed).

#### PECARN Experience

**Figure 3:**
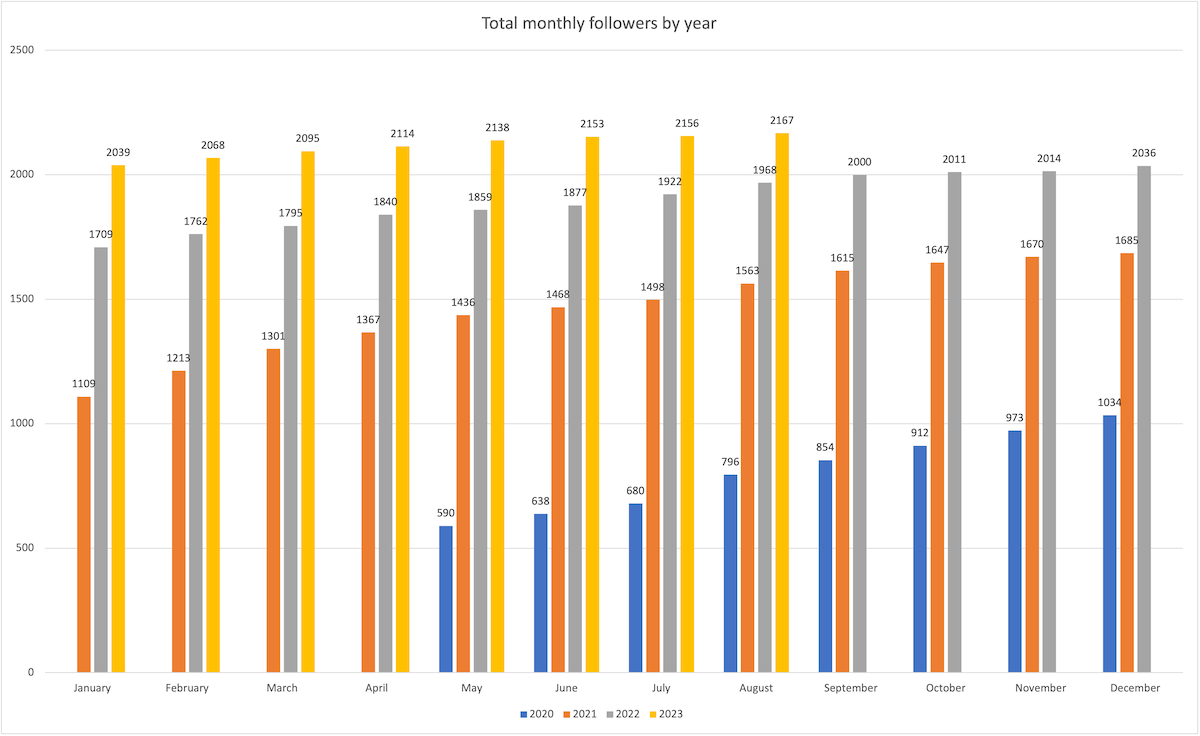
Total number of @PECARNteam Twitter account followers by month (May 2020-August 2023).

We focused primarily on trending the follower count, which grew consistently on a monthly basis (Figure 3). In April 2021 and later October 2022, we analyzed follower demographics to assess if we were reaching our target audience (Table 3). Emergency physicians comprised 47.7% (636/1332; April 2021) and 48.6% (927/1908; October 2022) of the followers. EMS providers consistently represented a small fraction of followers, accounting for only 1.7% (22/1332; April 2021) and 1.2% (23/1908; October 2022).

**Table 3:** Demographic distribution of the @PECARNteam Twitter account followers calculated in April 2021 and October 2022.

Twitter follower demographic			Year 2021 (n=1332), n (%)		Year 2022 (n=1908), n (%)
Health care worker					
	All physicians	933 (70.0)		1351 (70.8)	
	Emergency physicians^a^	636 (47.7)		927 (48.6)	
	Emergency medical services providers	22 (1.7)		23 (1.2)	
	Other health care workers^b^	83 (6.2)		137 (7.2)	
Non–health care workers			62 (4.7)		30 (1.6)
Medical organizations			76 (5.7)		116 (6.1)
Unidentified			156 (11.7)		251 (13.2)
Based in the United States			793 (59.5)		1158 (60.7)

^a^Emergency physicians included pediatric emergency physicians, general emergency physicians, and emergency medicine residents.

^b^Other health care workers included nurses, advanced practice providers, and medical students.

### Outcomes: Journal Article Metrics

#### Recommendation

Bibliometric data, such as the Altmetric Attention Score, should also be considered for tweets highlighting journal publications. In conjunction with traditional metrics such as journal publication citation counts, this score may serve as a complementary bibliometric and more real time, multisource reflection of online reach and attention.

#### PECARN Experience

Our 9-month exploratory analysis of the pre-post Altmetric Attention Scores demonstrated a median positive increase of 4.0 (range 0-24, SD 5.7) for the 41 consecutively featured PECARN journal publications (Multimedia Appendix 2). All but one of the tweets mentioning a journal article’s DOI or PubMed URL were associated with a nonzero increase in score. The single exception case resulted in the same pretweet and posttweet Altmetric score of 8.

## Discussion

### Principal Findings

Recent trends in academia and funding priorities emphasize the need for research dissemination extending beyond traditional journal publications, with a focus on reaching target audiences directly. Twitter and other social media platforms have emerged as potential tools to raise clinician awareness of the latest research. Unfortunately, there is limited literature to provide guidance for research organizations and medical institutions wishing to create an effective research dissemination campaign using social media [7,8,12,13].

This study presents a comprehensive roadmap for research organizations and medical institutions aiming to establish an effective Twitter presence for research dissemination. This roadmap, developed through a mixed methods approach, combining qualitative insights from our 4-year experience and quantitative data analysis, offers a structured framework in the form of a logic model. Our approach was particularly well-suited to the PECARN organization, which releases numerous practice-changing publications each year, such as the PECARN pediatric head trauma clinical decision tool [39]. This prolific output of high-quality studies allowed us to feature 99 unique publications over the study period, providing a rich dataset for our analysis and framework development.

The logic model we propose illustrates a complex, interconnected structure for organizations hoping to launch and maintain a social media account. Key inputs include organizational support, dedicated personnel, and technical resources. Our experience underscores the importance of a diverse team—including content writers, peer reviewers, account monitors, and graphic designers—to create and maintain a professional social media presence. The model also highlights the significance of consistent, high-quality content creation and strategic audience engagement as essential outputs. Importantly, our framework recognizes the intricate relationships between these elements, where inputs influence outputs, which in turn affect outcomes. These outcomes, such as engagement metrics and follower growth, provide valuable feedback that informs iterative refinement of our strategies. This continuous loop of implementation, assessment, and adjustment helps ensure adaptability in the dynamic social media landscape.

Our quantitative analysis yielded several key insights supporting the logic model framework, with impressions and engagement serving as our primary tweet-level outcome measures. Multiple regression analysis examining 6 tweet characteristics demonstrated meaningful explanatory power for both metrics.

Furthermore, 3 tweet elements—polls, graphics, and URL links—showed a stronger and statistically significant relationship with both impressions and engagements. The inclusion of polls demonstrated the strongest positive relationship, suggesting their effectiveness in stimulating audience attention and participation. This corroborates marketing strategies and recommendations [40,41]. This could be attributed to the ease of anonymous participation, the opportunity to engage with potentially controversial topics, and the instant gratification of immediate results. Graphics also showed a significant positive impact, likely due to their visually appealing nature and alignment with Mayer’s [42] multimedia principle, which states that people learn better from words and visuals juxtaposed together. This particularly reinforces the importance of including a graphic designer on the social media team, as outlined in our logic model. The inclusion of URL links, usually to journal articles, was also impactful, likely because it provided a primary reference for critical appraisal.

Interestingly, our analysis revealed that emojis had a negative association with impressions, contradicting some previous findings in social media research [38]. This may reflect the relatively small sample size of the regression analysis or possibly even the preferences of our specific audience of health care professionals, who might favor a more formal tone in academic communications. This suggests that strategies applicable in general social media marketing may not always apply to professional and academic contexts.

Thus, our current recommended tweet structure is to prioritize the inclusion of at least 3 tweet characteristics (polls, graphics, and URL links) to full articles or resources. Despite hashtags and account tagging being weakly significant in our study, current marketing recommendations support their use [43-45]. These 2 characteristics should be considered for inclusion depending on one’s audience, content, and organizational priorities. Hashtags and account tagging specifically may garner the attention of a community, field expert, or influencer who can amplify tweets to new audiences. Emojis could also be incorporated cautiously after careful consideration of the target audience given previous research supports their use. The evolution of the tweet structure should be informed by continued monitoring of Twitter analytics.

Our regression model accounted for 22%-26% of the outcome variance. Although this may seem a very modest association, it actually represents substantial explanatory strength within social science research contexts. Digital engagement patterns on platforms like Twitter are inherently influenced by numerous factors outside researcher control—algorithmic content prioritization, fluctuating user attention spans, competing real-world events, and platform-specific interaction dynamics. For context, established health communication and social science research typically considers *R*^2^ values of ≥0.1 as meaningful, recognizing that no statistical model can fully capture the complex interplay of individualized preferences, temporal factors, and contextual variables driving social media engagement behaviors [46].

Our *R*^2^ values indicate that while specific tweet characteristics contribute significantly to performance, additional factors beyond our model—such as content relevance, subject matter alignment with audience interests, and timing—may also influence tweet success. This finding reinforces the cross-disciplinary principle that “content is king” in digital communication environments. Our steady follower growth throughout the 4-year study period validates this content-focused strategy. Importantly, our follower demographics reveal that emergency physicians constitute nearly half of our audience, confirming an effective reach of our targeted professional community and suggesting a successful implementation of our dissemination approach.

We performed a 9-month, nonrandomized, exploratory, descriptive analysis of the Altmetric Attention Score for 41 consecutively featured journal articles, to confirm the Altmetric website’s assertion that a Twitter mention is part of their composite formula [43]. We reasoned that if any article showed no change in Altmetric score following our tweet activity, it would cast doubt on the reliability of Altmetric scores as a long-term outcome measure for our Twitter dissemination efforts. The observed increases in scores across all but one of the articles reinforced our confidence in using Altmetric data as one component of our evaluation strategy. Although the analysis was not designed to demonstrate a direct causal link, this analysis of both new and old PECARN publications supports Altmetric’s attestation. However, more research needs to be done to determine the impact of tweets on Altmetric scores.

Our proposed logic model for research dissemination has significant implications for knowledge translation in the digital age. Knowledge translation, often described as bridging the “know-do gap,” is the process of moving research findings into practical application and remains a significant challenge in health care [47,48]. Our findings align well with established frameworks in this field, particularly the knowledge to action (KTA) model and Rogers’ diffusion of innovations theory [49,50].

The KTA model comprises 2 main components—knowledge creation and action cycle. Knowledge creation involves knowledge inquiry, synthesis, and the development of knowledge tools [49]. The action cycle includes identifying problems, adapting knowledge to a local context, assessing barriers, implementing interventions, monitoring knowledge use, evaluating outcomes, and sustaining knowledge use. Our Twitter-based approach addresses several of these components. It facilitates knowledge synthesis and tool development by presenting research findings in concise, visually appealing formats easily accessible to clinicians. The consistent growth in followers and engagement metrics we observed suggests that this method effectively supports the knowledge-creation phase. In addition, our approach contributes to multiple steps in the action cycle. The elements we used, such as polls, further support the implementation and monitoring stages of the action cycle by actively engaging health care professionals.

Our study also aligns with the diffusion of innovations theory, which describes how new ideas spread through social systems [50]. Twitter, as a social media platform, acts as a channel for diffusion, allowing for rapid dissemination of research findings. The elements we used, such as polls, align with the theory’s emphasis on communication channels and social systems in the adoption of innovations. These elements potentially accelerate the diffusion process by engaging health care professionals and increasing their exposure to new research findings, thus facilitating the “knowledge” and “persuasion” stages of the innovation-decision process.

The effectiveness of visual content in our tweets underscores the crucial role of graphics in capturing attention, conveying complex information accurately, and summarizing content succinctly. This addresses a core principle in both the KTA model and diffusion of innovations theory—the need to present information in a clear, easily understandable format to promote its adoption. By translating complex research findings into digestible visual formats, we can potentially lower the barriers to adoption and increase the likelihood of research influencing clinical practice.

Several areas warrant further investigation. Longitudinal studies are needed to assess the long-term impact of social media dissemination on clinical practice changes. Exploration of other social media platforms for research dissemination could provide valuable comparisons to Twitter’s effectiveness. In addition, investigating the optimal frequency and timing of posts for maximum engagement among health care professional audiences could further refine social media strategies.

In summary, our logic model and mixed method findings provide a foundational framework for organizations seeking to incorporate Twitter and potentially other social media platforms for research dissemination. While our study demonstrates the potential of this approach, it also highlights the complexities involved in effective social media engagement for academic purposes. As the digital landscape continues to evolve, ongoing research and adaptation will be needed to optimize the dissemination of research findings to health care practitioners.

### Limitations

The methodology of our study faced certain limitations. While our logic model was crafted using qualitative and quantitative data with the PECARN Twitter account, there remains a possibility that critical elements for launching a professional Twitter account might have been overlooked. However, during our multiyear experience, we regularly received multisource feedback, likely identifying major challenges and uncovering hidden weaknesses.

Our experience was significantly influenced by 2 major external factors, potentially limiting the generalizability of our findings and recommendations. First, we launched our campaign just as the COVID-19 pandemic started in 2020. This period potentially enhanced our account’s visibility because of the shift toward social distancing and more digital connectivity. Second, since the time of our study, Twitter has since been sold and rebranded as X. It is unclear how the new leadership of Twitter will impact academic social media going forward and if the platform will continue in the same manner as during the study period. If Twitter were to be replaced with a new platform for professional discourse among health care professionals, our logic model principles can likely be adapted for other platforms.

Traditionally, the NGT is conducted in person, but due to the locations of our participants, we had to adapt to an online approach. Although a precedent exists in the literature, this shift to a remote format may have influenced the dynamics and outcomes of the discussions [51,52]. In our study, we chose to exclude the rank-order component of the NGT, opting instead to classify the events and insights based on their relationships to one another. This decision was because different elements carry different weights at different points in time when launching a professional social media account, thereby making ranking in order of importance irrelevant for our purposes.

Finally, 1 author (ML), the founder of the ALiEM organization (which was funded by the PECARN organization to manage the PECARN Twitter account), disclosed a potential conflict of interest. However, we mitigated this by having 2 external facilitators lead the entire NGT brainstorming session and identify the themes. Also, the collection and reporting of Twitter data was performed by the data analytics leader.

### Conclusion

Social media plays an increasingly vital role in research dissemination. Our initiative highlights the value of a deliberately designed Twitter presence in expanding the reach and awareness of a research organization’s scientific discoveries. Through practical experience, strategic planning, and data-driven iterative change, we propose a logic model roadmap designed to help other professional organizations in launching a social media campaign for research dissemination purposes.

## Appendix

Multimedia Appendix 1 Correlation coefficient matrix for tweet characteristics, impressions, and engagement.

Multimedia Appendix 2 Altmetric Attention Scores of 41 Pediatric Emergency Care Applied Research Network (PECARN) articles before and after tweet dissemination (August 2020-April 2021).

## Abbreviations

ALiEM: Academic Life in Emergency Medicine
DOI: Digital Object Identifier
EMS: emergency medical service
KTA: knowledge to action
NGT: nominal group technique
PECARN: Pediatric Emergency Care Applied Research Network

## Data Availability

All data analyzed during this study are included in the main manuscript and supplementary appendices of this publication, except for Twitter analytic data (available from the corresponding author upon request).
