# Supplementary material for: Research Dissemination Strategies in Pediatric Emergency Care Using a Professional Twitter (X) Account: A Mixed Methods Developmental Study of a Logic Model Framework
Source: JMIR Form Res. 2025 Jun 24;9:e59481. doi: 10.2196/59481 (PMC12238784; doi:10.2196/59481)
Supplement: Multimedia Appendix 1 [file formative_v9i1e59481_app1.docx]

| **Variable** | **Hashtag** | **Emoji** | **Poll** | **Graphic** | **Tagging** | **Link** | **Impressions** | **Engagement** |
| --- | --- | --- | --- | --- | --- | --- | --- | --- |
| Hashtag | 1 |  |  |  |  |  |  |  |
| Emoji | 0.068 | 1 |  |  |  |  |  |  |
| Poll | -0.192 | -0.124 | 1 |  |  |  |  |  |
| Graphic | 0.129 | 0.001 | -0.137 | 1 |  |  |  |  |
| Tagging | 0.252 | 0.045 | -0.165 | 0.214 | 1 |  |  |  |
| Link | 0.276 | -0.087 | -0.078 | 0.214 | 0.317 | 1 |  |  |
| Impressions | 0.178 | -0.095 | 0.158 | 0.252 | 0.094 | 0.231 | 1 |  |
| Engagement | 0.161 | -0.065 | 0.275 | 0.267 | 0.056 | 0.231 | 0.834 | 1 |
