# Supplementary material for: Research Dissemination Strategies in Pediatric Emergency Care Using a Professional Twitter (X) Account: A Mixed Methods Developmental Study of a Logic Model Framework
Source: JMIR Form Res. 2025 Jun 24;9:e59481. doi: 10.2196/59481 (PMC12238784; doi:10.2196/59481)
Supplement: Multimedia Appendix 2 [file formative_v9i1e59481_app2.docx]

Appendix 2: Altmetric Attention Scores of 41 PECARN articles before and after tweet dissemination (August 2020-April 2021)

| Article # | Pre-Tweet Altmetric Score | Post-Tweet Altmetric Score | Post-Pre Difference | % Increase |
| --- | --- | --- | --- | --- |
| 1 | 68 | 88 | 20 | 29.4% |
| 2 | 2 | 10 | 8 | 400.0% |
| 3 | 33 | 45 | 12 | 36.4% |
| 4 | 17 | 28 | 11 | 64.7% |
| 5 | 1 | 8 | 7 | 700.0% |
| 6 | 349 | 370 | 21 | 6.0% |
| 7 | 1 | 6 | 5 | 500.0% |
| 8 | 38 | 47 | 9 | 23.7% |
| 9 | 17 | 21 | 4 | 23.5% |
| 10 | 1 | 6 | 5 | 500.0% |
| 11 | 130 | 135 | 5 | 3.9% |
| 12 | 3 | 6 | 3 | 100.0% |
| 13 | 1 | 5 | 4 | 400.0% |
| 14 | 2 | 9 | 7 | 350.0% |
| 15 | 4 | 8 | 4 | 100.0% |
| 16 | 8 | 8 | 0 | 0.0% |
| 17 | 180 | 185 | 5 | 2.8% |
| 18 | 2 | 6 | 4 | 200.0% |
| 19 | 11 | 16 | 5 | 45.5% |
| 20 | 42 | 43 | 1 | 2.4% |
| 21 | 92 | 99 | 7 | 7.6% |
| 22 | 21 | 45 | 24 | 114.3% |
| 23 | 16 | 19 | 3 | 18.8% |
| 24 | 4 | 5 | 1 | 25.0% |
| 25 | 1 | 3 | 2 | 200.0% |
| 26 | 151 | 153 | 2 | 1.3% |
| 27 | 2 | 6 | 4 | 200.0% |
| 28 | 14 | 20 | 6 | 42.9% |
| 29 | 3 | 6 | 3 | 100.0% |
| 30 | 3 | 14 | 11 | 366.7% |
| 31 | 31 | 34 | 3 | 9.7% |
| 32 | 2 | 12 | 10 | 500.0% |
| 33 | 13 | 15 | 2 | 15.4% |
| 34 | 5 | 16 | 11 | 220.0% |
| 35 | 4 | 7 | 3 | 75.0% |
| 36 | 919 | 920 | 1 | 0.1% |
| 37 | 3 | 6 | 3 | 100% |
| 38 | 887 | 890 | 3 | 0.3% |
| 39 | 3 | 5 | 2 | 66.6% |
| 40 | 0 | 19 | 19 | n/a |
| 41 | 14 | 18 | 4 | 28.6% |
